# Does a Father's Social Environment Influence Their Sons' Sperm Sex Ratio? Potential for the Epigenetic Transmission of a Sex‐Allocating Mechanism

**DOI:** 10.1002/ece3.72519

**Published:** 2025-12-26

**Authors:** Renée C. Firman, Francisco Garcia‐Gonzalez

**Affiliations:** ^1^ Centre for Evolutionary Biology, School of Biological Sciences (M092) The University of Western Australia Crawley WA Australia; ^2^ Estacion Biológica de Doñana CSIC Sevilla Spain

**Keywords:** house mice, intergenerational inheritance, paternal effect, sex allocation, social environment

## Abstract

Recent investigations have demonstrated that males exposed to other males during development produce lower numbers of Y‐chromosome bearing sperm. Despite the potential for legacy effects, the multigenerational implications of variation in the paternal social environment for the sperm sex ratio have not been investigated. Here, we exposed male house mice (fathers) to high‐male or high‐female density conditions during their sexual development and quantified the sperm sex ratio of their sons. Our analysis revealed that the sons of fathers reared under high‐male density conditions, produced, on average, higher numbers of daughter‐producing sperm compared to sons of fathers reared under high‐female conditions. As environmental and genetic influences in sons were controlled for (common‐garden breeding and family‐based design), this result can be attributed to nongenetic inheritance. Although our experiment produced a significant result, we acknowledge that the difference in the sons' sex ratio was small and that further investigation with the application of a more sensitive sperm sex ratio quantification method may produce a more robust outcome. Nevertheless, our investigation demonstrates the potential for the intergenerational transmission of the sperm sex ratio. We discuss the intergenerational nature of the sperm sex ratio as an adaptive strategy for increasing paternal fitness within different social environments and highlight mechanisms that could account for this result.

## Graphical abstract

We exposed male house mice (fathers) to high‐male or high‐female density conditions during their sexual development and quantified the sperm sex ratio of their sons. Our analyses revealed that the sons of fathers reared under high‐male density conditions, produced, on average, higher numbers of daughter‐producing sperm compared to sons of fathers reared under high‐female conditions. Our investigation demonstrates the potential for the intergenerational transmission of the sperm sex ratio.

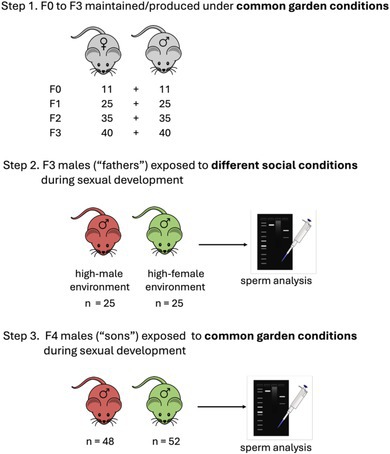

## 1 ∣Introduction

It has been recognised for many years that the inheritance of genetic information (variations in DNA sequence) is accompanied by additional epigenetic marks (Perez and Lehner 2019). As scientists continue to examine the dynamic mechanisms of epigenetic inheritance (e.g., histone modifications, DNA methylation and noncoding RNAs) through advances in molecular methods, our understanding of how epigenetic modifications occur in germlines and are transmitted to the following generation continues to grow (Bell and Hellmann 2019). We now know that in mammals there are two major reprogramming events that erase and replace existing epigenetic marks; during germ cell development and during the preimplantation period of embryo development (Bell and Hellmann 2019). Epigenetic modifications that endure both reprogramming events become integrated and persist throughout the life of the individual (within‐generation plasticity). Moreover, these epigenetic marks may be passed down to the next generation (intergenerational plasticity). The parental environment can thus lead to long‐term consequences affecting the phenotype and survival of future generations.

Maternal transmission represents the most obvious route by which parents can influence offspring phenotype independently of the transmission of DNA sequences. The intimate relationship between mother and young during development makes this especially true for mammals and is likely the reason that maternal effects have long been the focus (Mousseau and Fox 1998). Fathers, however, can also be conduits of parental effects and transmit information to their offspring via the sperm epigenome and other ejaculate constituents outside of sperm DNA sequence transmission (Crean and Bonduriansky 2014; Evans et al. 2019). It has become increasingly clear that sperm and ejaculate epigenetic information from fathers can influence the adult phenotype of their progeny in a multi‐generational capacity (Chen et al. 2016; Immler 2018; Tyebji et al. 2020). For example, smoking in humans is known to alter sperm DNA methylation patterns and negatively affect offspring health (Liu et al. 2022). In addition to toxicants, however, paternal effects may arise under variation in the exposure of fathers to different environments, stressful events or social factors (Evans et al. 2019).

When it comes to offspring sex, the social environment can have profound implications for parental fitness strategies. For example, sex allocation theory states that producing sons under high‐male density conditions is disadvantageous because sons will be required to compete with unrelated local resident males, as well as nondispersing brothers, for access to territories and/or mates (Hamilton 1967). Further, for polyandrous species or populations, high‐male density conditions often translate to intense postmating male–male competition (Firman 2020b; Parker 2000). In contrast, females will not be forced to compete. Rather, with high‐male density daughters are guaranteed mate availability and are therefore valued as the favoured sex (Hamilton 1967). Research on species that exhibit direct maternal control over offspring sex, such as spider mites, has demonstrated how mothers respond to the social environment and skew offspring sex ratios in a manner that will maximise fitness (Macke et al. 2012; Roeder 1992).

But it is not only mothers that are able to influence the sex of their young, and for mammals male‐driven sex allocation has recently emerged as an important area of research (Douhard 2018; Edwards and Cameron 2014; Malo et al. 2017). Male house mice are highly territorial and continually deposit urinary volatiles to deter competitors from their area (Hurst and Beynon 2004), and thus are a promising system for testing anticipatory parental responses based on changes or variation in the social environment (Whitman and Agrawal 2009). Empirical investigations on male house mice have focused on adjustments in the sperm sex ratio $\left(n_{Y-\text{chromosome bearing sperm}\;\left(Y-\mathrm{CBS}\right)}/n_{\text{total sperm}}\right)$ in response to the social environment, for example whether or not they are exposed to male competition during development (Firman et al. 2020; Lavoie et al. 2019). This strategy is expected to be beneficial as males could reduce son production when male density is high, reducing the level of sexual competition that their male offspring would be exposed to, and favouring daughter production, which would yield the highest fitness returns. Social conditions could also define sex allocation responses in subsequent generations. For example, if density conditions are typically relatively stable (i.e., not highly fluctuating across generations), such intergenerational plasticity would be adaptive.

It is not yet known how paternal exposure to male competition during development influences the sperm sex ratio of the offspring. In this investigation, we studied house mice to address this outstanding question. We reared males (fathers) in either a high‐male or high‐female environment and analysed the sperm sex ratios of their offspring (sons). Genetic influences were controlled by using brothers across treatments and sons were treated identically during sexual development by being reared under common‐garden conditions, which allows us to draw conclusions about the nongenetic inheritance of the sperm sex ratio.

## 2 Materials and Methods

### 2.1 Experimental Model and Common‐Garden Breeding

**FIGURE 1:**
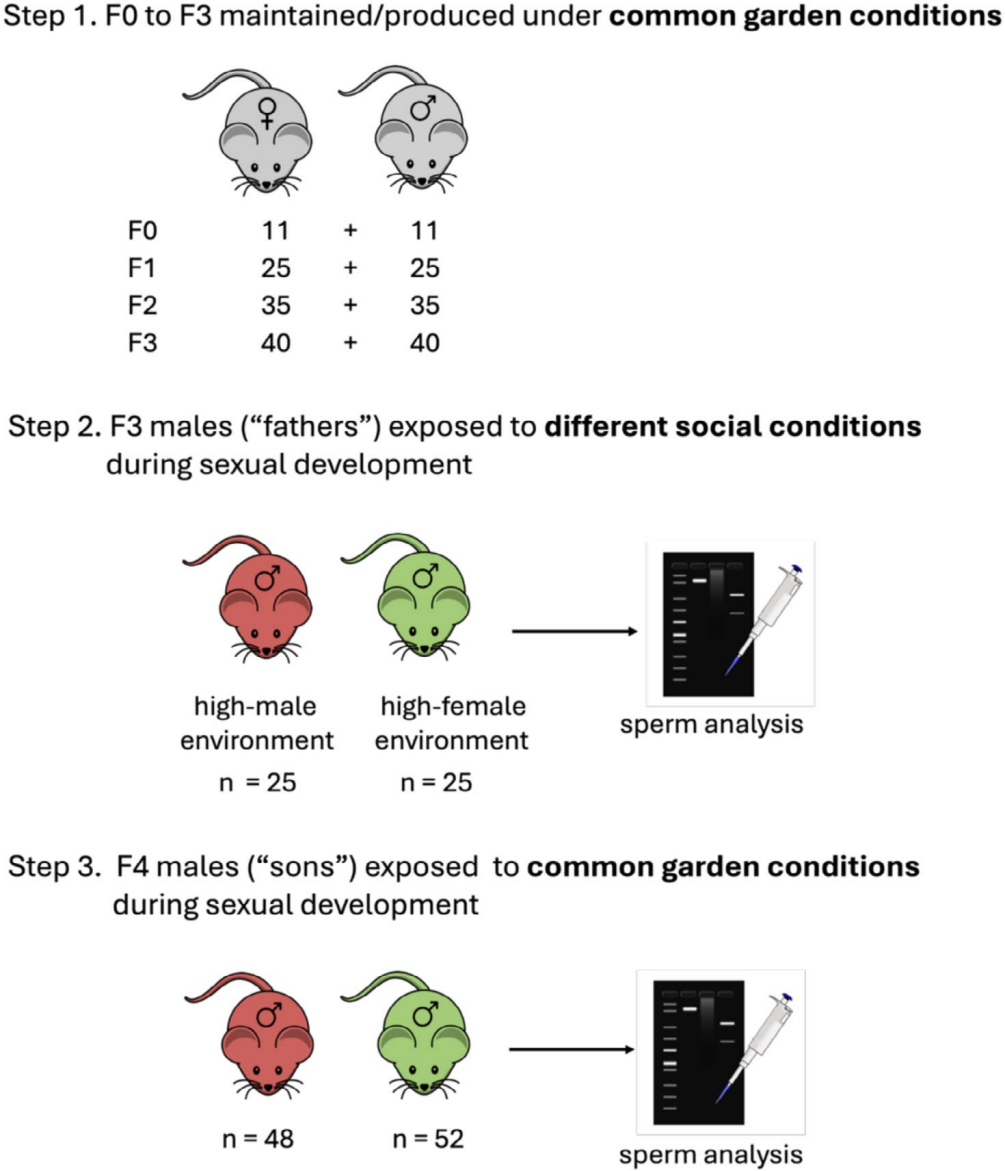
Stepwise overview of the experimental design. Wild‐caught house mice (F0) were captured and transported to the University of Western Australia. The mice were outbred for two generations (F1, F2) under common‐garden conditions to eliminate potential environmental factors that may affect the sperm sex ratio. Step 1 includes the numbers of animals that contributed to each generation. ‘Fathers’ (F3) were exposed to different social environments using established protocols known to induce phenotypic plasticity in the reproductive traits of house mice (see Figure S1 for details). As the target sample size of ‘sons’ was 50 males per treatment, five extra ‘fathers’ were established per treatment to account for reproductive failure. ‘Fathers’ were sourced from families such that a pair of brothers was used across treatments. The brother pairs were mated with a pair of sisters to produce the ‘sons’ (F4). The sons developed under common‐garden conditions. Sperm from both the ‘fathers’ (F3) and ‘sons’ (F4) was extracted and analysed. Steps 2 and 3 include the ‘fathers’ (target) and ‘sons’ (actual) sample sizes.

Wild house mice ( *Mus musculus domesticus* ; *n* = 22) were captured from Boullanger Island, located off the coast of Western Australia (30°18′55″ S, 115°00′13″ E), and transported to the University of Western Australia. To control for environmental influences on the traits under investigation (Figure 1), mice were maintained and bred under standardised common‐garden conditions for two generations (F1, F2). They were housed in standard cages (16 × 33 × 12 cm) at a constant temperature of 24°C on a reverse 14:10 light–dark cycle. All animals received food and water *ad libitum* and were subject to the same husbandry procedures.

Mating occurred during the dark phase under red lighting, following the methods of (Firman and Simmons 2008). Virgin, sexually mature females were monitored regularly to detect oestrus (Byers et al. 2012). Upon confirmation of oestrus, females were introduced into male cages, and monitored every 30 min for the presence of a mating plug, which was used as an indicator of successful copulation (Firman and Simmons 2008). After mating, females were transferred to clean cages with shredded paper for nesting and left undisturbed until giving birth. Offspring were weaned at 3 weeks of age, at which point the experimental males (= fathers) were exposed to different social environments as detailed below.

### 2.2 Social Environment Manipulation (Fathers) and Common‐Garden Rearing (Sons)

We exposed male mice (fathers; F3) to distinct social environments using established protocols known to elicit phenotypic plasticity in reproductive traits in house mice (André et al. 2018; Firman 2020a; Firman et al. 2023, 2018, 2013; Firman and Simmons 2013; Lavoie et al. 2019). While maintaining a consistent overall density of individuals, we manipulated fathers' exposure to male and female pheromonal cues (Firman 2020a; Lavoie et al. 2019). Fathers were assigned to either a high‐male (*n* = 40) or high‐female (*n* = 40) social environment (Figure 1). To control for genetic and family‐derived variation, brothers from the same litter were split between the two treatments.

The social environments were established by housing males in standard cages arranged on metal racks across two separate constant temperature rooms (Figure 1). In the high‐male condition, males were reared from three to 15 weeks of age in proximity to other males, including both age‐matched individuals and sexually mature males. Cage positions were regularly rotated within the room to ensure uniform exposure. Twice weekly, each male was given 15 g of soiled bedding (chaff) collected from 16 sexually mature males. Additionally, once every two weeks, each male participated in a ‘male encounter’. During these encounters, the focal male was placed in a large opaque plastic tub (49 × 74 × 41 cm) containing two sexually mature males housed inside their home cages. For 30 min, the focal male was allowed to roam freely within the tub, interacting indirectly with the caged males through the wire cage lids. Different males were used in each encounter to ensure a range of social stimuli. To support normal reproductive development, focal males were also periodically exposed to soiled chaff from a sexually mature female.

The high‐female environment was established in a second constant temperature room, where males were reared from three to 15 weeks of age in proximity to females. These included both sexually mature females and age‐matched females (Figure 1). As with the high‐male treatment, cage positions were regularly rotated to ensure uniform exposure across the room. Twice per week, each male was provided with 15 g of soiled bedding (chaff) collected from 16 sexually mature females. In addition, males participated in fortnightly ‘female encounters’, following the same procedure used in the high‐male condition. During each encounter, the focal male was placed in an opaque plastic tub for 30 min with two sexually mature females housed in their home cages, allowing for indirect interaction through the wire cage lids. To control for potential room‐based environmental effects, the high‐male and high‐female treatments were switched between the two rooms midway through the experiment.

Fathers were maintained in the high‐male and high‐female social environments until reaching sexual maturity (~100 days of age), at which point they were mated to produce the next generation (F4). To control for family‐derived maternal variation, brothers were each mated with an unrelated female from the same family (i.e., sisters). These females were from the same generation as the males and had been reared under standardised, common‐garden conditions in a separate room from the experimental treatments.

Breeding was conducted under the same common‐garden protocol described above: (i) females were monitored regularly to detect oestrus (Byers et al. 2012); (ii) oestrus females were introduced into male cages to initiate mating; (iii) the presence of a mating plug was checked every 30 min as an indicator of successful copulation; and (iv) following mating, females were housed individually in clean cages with shredded paper and left undisturbed until parturition. Nine days after mating, fathers were euthanised via cervical dislocation, and sperm samples were collected for analysis (Firman et al. 2023).

F4 litters were weaned at three weeks of age. Male offspring (= sons) were housed individually and reared under common‐garden conditions until sexual maturity. Throughout this period, they received food and water *ad libitum* and experienced standardised husbandry practices. From weaning, sons were housed in rows consisting of four individually housed males flanked by two pairs of females (sisters when possible), ensuring each male was adjacent to one pair of females. To minimise positional bias, male cages were rotated every fortnight in a randomised order while maintaining the overall rack configuration.

Once sons reached sexual maturity (~100 days of age), they were euthanized via cervical dislocation. These experimental subjects were originally used to test whether developmental social environments influence intergenerational inheritance of sperm competition traits (see Firman et al. 2023). For the present study, sperm were extracted and stored for subsequent analysis as described below.

### 2.3 Sperm Isolation and DNA Extraction

Following euthanasia, the epididymides of males were removed and incised in human tubal fluid (HTF; 1 mL), and incubated (37°C, 5% CO_2_; 10 min) to allow the sperm to swim into the medium. After this, the tissue was removed and the suspension was incubated for 50 min. Sperm quantity was quantified using the CEROS II computer‐assisted sperm analysis system under standard mouse sperm parameters (version 1.3, Hamilton Thorne Research) by loading ∼10 μL of sperm suspension into a haemocytometer and scanning 10 fields of view. The mean sperm number value of the 10 replicate scans was used in the analyses. The remaining sample was centrifuged (10 min; 14,000 rpm), washed in Tris‐EDTA (500 μL) and recentrifuged (5 min; 14,000 rpm) to pellet the sperm. Finally, sperm were resuspended in Tris‐EDTA (200 μL) and stored (−20°C).

We extracted genomic DNA from pooled sperm samples by Chelex‐100 as we have done previously (Lavoie et al. 2019; Silva et al. 2014). Briefly, a 25 μL aliquot of thawed sperm solution was added to Chelex‐100 resin buffer (200 μL; 5%) and digested with Proteinase K (20 μL; 20 mg/mL) and DTT (7.6 μL; 31 mM) (incubation 45mins; 56°C). Following a Proteinase K enzyme deactivation period (8mins; 95°C), the samples were centrifuged (3mins; 10,500 rpm) and the supernatant was quantified by a spectrophotometer. All samples were standardised to [100 ng/μL] by dilution with nuclease‐free water and stored (−20°C) prior to quantitative real‐time polymerase chain reaction (qPCR).

### 2.4 Sperm Genotyping

We quantified the proportion of Y‐CBS using an absolute quantification qPCR protocol previously optimised and validated for mouse sperm samples (Firman et al. 2020; Lavoie et al. 2019). Briefly, DNA was amplified in triplicate 10 μL singleplex reactions using a standardised concentration of 100 ng/μL, targeting the *G6pd2* (X‐linked) and *Sry* (Y‐linked) genes. Reaction components and thermal cycling conditions followed those detailed in (Lavoie et al. 2019). Fluorescence was measured at the end of each amplification cycle to determine the threshold cycle (Ct) values.

For each sperm sample, the mean Ct values from triplicate reactions for *G6pd2* and *Sry* were used to calculate the proportion of X‐ and Y‐CBS following the method described by (Parati et al. 2006; Puglisi et al. 2006). The proportion of Y‐CBS was derived using the equation:

$$\text{proportion}\;Y-\mathrm{CBS}=n/\left(n+1\right)$$

where *n* = Ct_Y‐CBS_/Ct_X‐CBS_.

The total number of X‐ and Y‐CBS in each sample was then calculated based on (i) the proportion from the qPCR assay, (ii) the volume of sperm suspension used in the assay and (iii) the sperm concentration measured at the time of sperm collection.

### 2.5 Statistical Analyses

Variation in the sperm sex ratios of the sons (*n*_sons_ = 100) was analysed using the *glmer* function implemented in the package *lme4* version 1.1.35.1 (Bates et al. 2015). We fitted a generalised linear mixed model (GLMM) with a binomial error distribution using the command *cbind* to take into account the numbers of X‐ and Y‐CBS leading to a ratio for each sample. Family ID (31 families) and Father ID (47 fathers) were included as random factors in a model with social condition (two levels) as a fixed factor. The model was underdispersed, and the standard errors of the estimates in this model were corrected by the dispersion factor to recompute *Z* and *p* values. As a confirmatory measure, and because all datapoints of Y‐CBS proportion were based on high total numbers of sperm across the two social conditions (range for total number of sperm: 141–751 for high‐male density, 105–881 for high‐female density), we ran a LMM using the same fixed and random structure as above. Hypothesis testing in the LMM was carried out through a Wald chi‐squared test on a maximum likelihood model using the function *Anova* in the package *car* 3.0.13 (Fox and Weisberg 2019). In addition to that, the significance of the fixed effect was assessed with a Wald F‐test on a restricted maximum likelihood (REML) model using the package *lmerTest* 3.1.3 (Kuznetsova et al. 2017). The estimates were calculated using REML.

Effect sizes (Cohen's *d*) and associated 95% CIs were calculated with bootstrapping using the package *emmeans* version 1.8.8 (Lenth 2022), on marginal means from the LMM model. Model diagnostics were carried out by calculating and plotting scaled (quantile) residuals using the package ‘DHARMa’ (Hartig 2022). Analyses were run in R version 4.2.0 (R Core Team 2022).

## 3 Results

**FIGURE 2:**
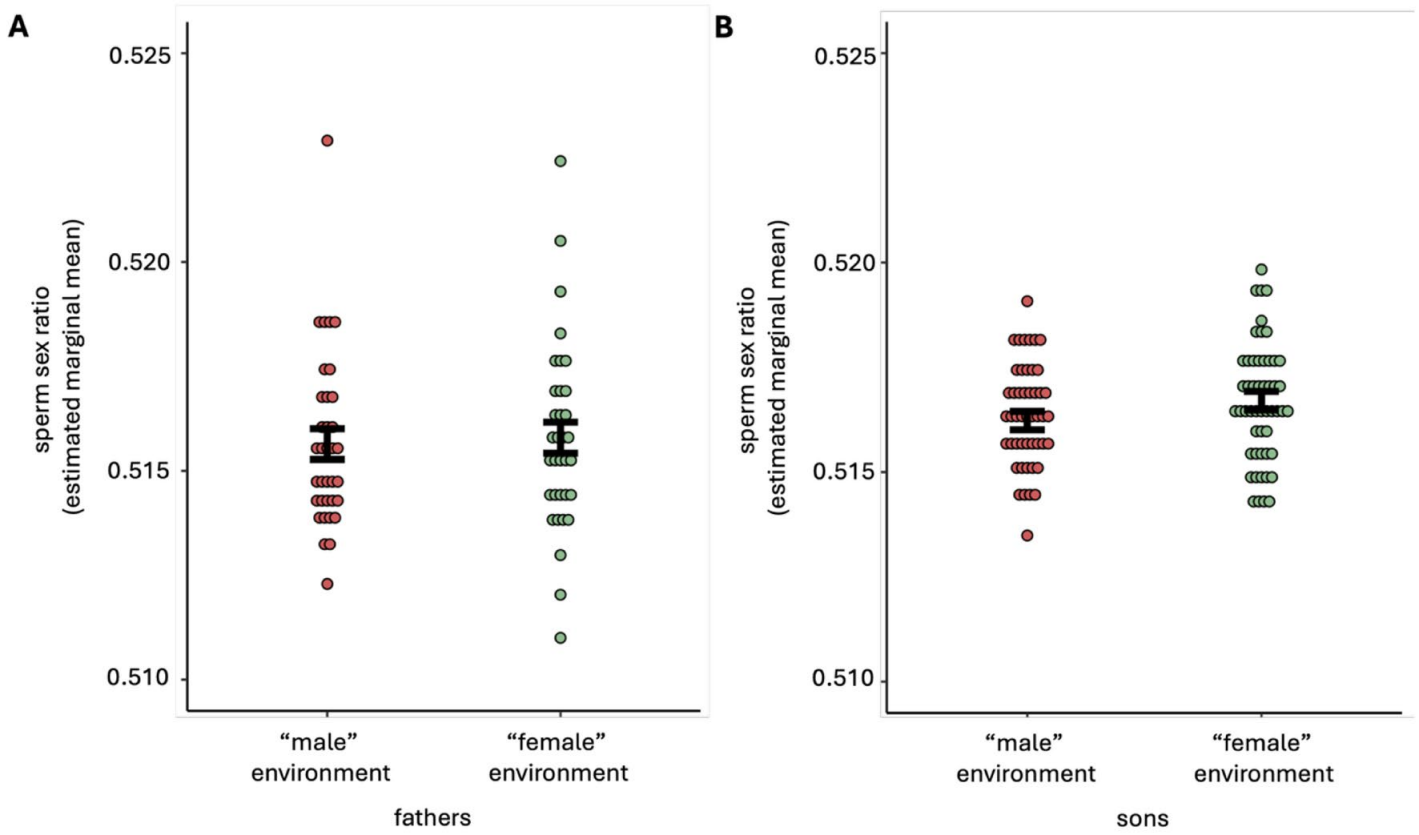
Estimated marginal means and associated standard errors (black dots with error bars) of the sperm sex ratios (proportion Y‐CBS) of (A) ‘fathers’ exposed to high‐male or high‐female density social conditions during development and (B) the ‘sons’ exposed to common‐garden conditions during development.

The results were consistent with the existence of a treatment effect on the sons' sperm sex ratio. The binomial model, as well as the Wald chi‐squared test from a LMM model, supported the existence of a significant paternal social environment effect such that males reared in a high‐male density environment produced sons with lower proportions of Y‐CBS than the sons of males reared in a high‐female density environment (Table 1, Table 2, Figure 2). The Wald F‐test from the LMM returned a marginally nonsignificant outcome, reflecting uncertainty regarding the presence of a clear effect (Table 1, Figure 2). However, the analysis of the effect size suggests that a potentially strong influence should not be dismissed (Cohen's *d* = 0.44 [−0.02, 0.91]; Table 1). In contrast, we found no evidence for a significant effect of the social rearing environment on the sperm sex ratio of fathers (Table 2, Figure 2).

**TABLE 1:** Linear mixed models testing the difference in sperm sex ratios of (a) ‘fathers’ exposed to high‐male or high‐female density environments during development and (b) the ‘sons’ exposed to common‐garden conditions during development. The table includes the effect size estimate (Cohen's *d*) and its associated 95% confidence interval (CI).

	Fixed effect	Estimate	s.e.	Type II Wald *χ* ^2^	*p*	Cohen's *d*	95% CI
(a) Fathers	Intercept	0.51560	0.00037				
	Environment	0.00015	0.00050	0.096^a^	0.757	0.07	[−0.41, 0.56]
(b) Sons	Intercept	0.51620	0.00022				
	Environment	0.00048	0.00024	4.024^b^	**0.045**	0.44	[−0.02, 0.91]

*Note:* The *p* value in bold is significant at < 0.05.

^a^ Type II Wald F‐test with Kenward–Roger degrees of freedom for the environment effect in the fathers' model: F_1,33.682_ = 0.093, *p* = 0.763.

^b^ Type II Wald F‐test with Kenward–Roger degrees of freedom for the environment effect in the sons' model: F_1,19.887_ = 3.813, *p* = 0.065.

**TABLE 2:** Generalised linear mixed model (binomial error distribution) testing the difference in sperm sex ratios of (a) ‘fathers’ exposed to high‐male or high‐female density environments during development and (b) the ‘sons’ exposed to common‐garden conditions during development.

	Fixed effect	Estimate	s.d.	*Z*	*p*
(a) Fathers	Intercept	0.06406	0.00159		
	Environment	0.00001	0.00231	0.005	0.996
(b) Sons	Intercept	0.06499	0.00079		
	Environment	0.00294	0.00116	2.533	**0.011**

*Note:* The *p* value in bold is significant at < 0.05.

## 4 Discussion

The assessment of cues or stimuli can provide individuals with information that is predictive of future conditions. Intragenerational investigations on house mice have examined socially mediated phenotypic plasticity in the context of male–male competition (Firman et al. 2018, 2013; Ramm and Stockley 2009) and demonstrated that males reared in the presence of other males, or cues that signify competitor presence, develop a phenotype that is expected to be adaptive when engaging in physical combat (i.e., larger body size) or sperm competition (i.e., elevated sperm numbers) (Firman et al. 2018, 2013; Ramm and Stockley 2009). Moreover, a recent investigation showed that these responses persisted in the next generation via nongenetic inheritance (Firman et al. 2023). Here, we studied house mice to examine how the paternal social environment influences the sperm sex ratio in subsequently conceived offspring. In a highly controlled experimental setting where sources of variance attributable to differences between individuals and lineages were controlled, we found evidence that the sons of males reared in a high‐male density environment produced, on average, lower proportions of Y‐CBS than the sons of males reared in a high‐female density environment. There was discrepancy regarding the existence of significant treatment effects, depending on the test employed, when using the fixed *p* value threshold of 0.05 (Wald test *p* = 0.045 vs. *F*‐test *p* = 0.065). Importantly, however, a large effect size cannot be ruled out (Cohen's *D* = 0.44, [−0.02, 0.91]). Using a fixed *p* value threshold (i.e., 0.05) to interpret results can discourage further exploration of potentially important findings (Nakagawa and Cuthill 2007; Wasserstein et al. 2019). Our effect size calculations suggest that paternal social environment effects on offspring sperm sex ratio merit further study. Indeed, use of a sperm sexing assay that is more sensitive than the qPCR method applied here might yield a more robust effect of treatment. Previous studies on mice have utilized a range of methods to quantify the sperm sex ratio, including the use of X‐ and Y‐chromosome‐specific paint probes (Edwards et al. 2016) and flow cytometry (Welch and Johnson 1999). While these methods required specialised equipment and professional skills, more recent advancements in sexing sperm have focused on the functional differences between X‐ and Y‐CBS. An example of this takes advantage of X‐ and Y‐CBS differences in ligand activation on sperm toll‐like receptors 7/8 (TLR7/8) and the selective suppression of X‐CBS motility (without altering sperm viability or acrosome formation), while Y‐CBS motility remains uninhibited (Umehara et al. 2020). The difference in sperm motility results in the layered separation of X‐ and Y‐CBS in medium that can be collected and used to quantify the sperm sex ratio or for offspring sex selection (IVF) (Umehara et al. 2020). A more comprehensive understanding of the intergenerational nature of the sperm sex ratio may come from the application of this methodology. Nevertheless, we demonstrate here for the first time, the potential for the social environment experienced by fathers to affect the sperm sex ratio of their sons. Below we discuss the adaptive implications and explanatory mechanisms of the transmission of this result.

Most species produce equal numbers of sons and daughters (Clutton‐Brock and Iason 1986); however, the temporary overproduction of the scarcer sex may be adaptive under certain situations, for example, when parents can anticipate future mating opportunities (Fisher 1930; West 2009). High‐male density social conditions have been shown to lead to elevated stress hormone levels and female‐biased in utero offspring sex ratios in female house mice (Firman 2020a). Sex‐biased offspring production (prior to or during embryonic implantation), and not differential mortality, was identified to be the mechanism accounting for this skew (Firman 2020a). In alignment, our previous study demonstrated that male house mice experiencing intense male competition during development produced higher proportions of X‐CBS (Firman et al. 2020). Interestingly, we did not observe any difference in the sperm sex ratios of fathers subjected to different social environments in the current study. This difference is likely explained by variation in the experimental designs and the differences in the level of competition perceived by fathers during development (i.e., in our earlier study, males were reared under ‘competitive’ conditions by being contained in their own individual box but residing in close proximity to two other males inside a large plastic box) (Firman et al. 2020). The phenomenon of epigenetic information being transmitted intergenerationally without altering the paternal phenotype is commonly observed. An example of this is seen in the agouti mouse model; fathers fed a specific diet show no visible trait changes, but their sperm exhibit altered DNA methylation patterns and their offspring display changes in coat colour and other traits (Irmler et al. 2020). Similarly, a recent study on sheep demonstrated that 34 transgenerational methylated genes influenced by paternal nutrition ‘escape’ reprogramming and impacted the growth and development of the F1 and F2 offspring, including male fertility traits (Braz et al. 2023).

For fathers and sons alike, a bias in the production of daughters under high‐male density would lead to the avoidance of reproductive competition among sons and thus could be deemed an adaptive parental strategy (Hamilton 1967). However, it is not currently clear why selection would favour an adjustment in the offspring and not the individuals experiencing the environmental cues. Rapid changes in the social landscape can arise through variation in sex‐specific dispersal or mortality and differences in the extent that generations overlap (Werren and Charnov 1978) and therefore the environment experienced by the parent will not always be representative of the future conditions that the offspring will experience. Sex‐biasing mechanisms that are transferred to the next generation, as demonstrated here in the sperm sex ratio, thus have the potential to be adaptive, maladaptive or neutral. In the case that sons are faced with high‐male density conditions as their fathers had experienced, higher proportions of X‐CBS may lead to an adaptive outcome with a bias in the production of daughters. When the parent–offspring environments are mismatched, the production of daughters under high‐female density conditions would likely compromise reproductive fitness. An important aspect to consider is that here developmental conditions for sons were kept equal (common garden). Our result might therefore suggest that the intergenerational effect operates as an anticipatory response that is beneficial in principle, unless the conditions are reversed, in which case sons can also respond adaptively as observed in our previous study (Firman et al. 2020). Certainly, further investigation is required to understand the fitness implications of epigenetically inherited sperm sex ratios under different social conditions.

The mechanism(s) by which skewed sperm sex ratios arise is currently not well understood and we can only speculate on how the impact of the social environment on sperm sex ratios may be transferred intergenerationally. Epigenetics broadly encompasses mechanisms that lead to changes in gene expression without altering the underlying DNA sequence, which is likely to account for the result that we see here with sons expressing sperm sex ratios based on the social conditions that their fathers experienced during development. DNA methylation is one of the most extensively studied epigenetic modifications and plays a crucial role in various processes within the mammalian genome, including X chromosome inactivation, gene silencing, genomic stability and genomic imprinting (SanMiguel and Bartolomei 2018). Although DNA methylation is a stable and heritable epigenetic mark, it is also highly dynamic, especially during mammalian development (SanMiguel and Bartolomei 2018). However, other epigenetic mechanisms have also been implicated in the transmission of paternal effects, including oxidative damage to sperm DNA, histone modifications and changes in small noncoding RNA (Yehuda and Lehrner 2018). Early research on laboratory mice demonstrated how transgenerational effects can be inherited via parental gametes (e.g., [Dias and Ressler 2014]), and there is now compelling evidence of sperm‐mediated transmission and retention of paternal epigenetic marks in the embryo (Lismer and Kimmins 2023).

Potential mechanisms that could lead to unequal sperm sex ratios have been identified to be differential selective apoptosis or epididymal phagocytosis, or transmission ratio distortion (‘sex chromosome meiotic drive’) (Jaenike 2001; Saragusty et al. 2012). The latter seems doubtful as selfish drivers on sex chromosomes are unlikely sensitive to social environments and improbable that selection at the level of the host would induce potentially adaptive responses in driver expression. Skewed sperm sex ratios can also arise when an imbalance occurs as a dysfunction of spermatogenesis (Gellatly 2009; Pawluk and Zagalska‐Neubauer 2024). Although there is currently no evidence that one sperm type has a higher genesis error than another (Rahman and Pang 2020), there tends to be an association between impaired sperm quality and the production of daughters (Bae et al. 2017; Eisenberg et al. 2012; Gomendio et al. 2006). Sperm collection in mice involves competent motility so that cells can swim free of the epididymal tissue. Thus, differential ‘performance’ of sperm may potentially account for shifts in the X‐CBS to Y‐CBS ratio. Furthermore, stress experienced during development has been shown to lead to high proportions of morphologically abnormal sperm, but it is unknown whether this impairment influences sperm sex ratios and is transferred to the next generation (Du et al. 2024; García‐Vargas et al. 2019). Whatever the underlying mechanism, it seems that epigenetic marks acquired by fathers under specific social conditions are imprinted in the germline and subsequently reflected in the sperm sex ratios of their sons. It would be valuable for future research to explore potential mechanisms that may account for intergenerational transmission of the sperm sex ratio.

It was once only mothers that were considered to be the determiners of offspring sex ratio skews. In recent years, this view has been reassessed with the understanding that the sperm sex ratio is a variable trait that can govern offspring sex under certain conditions. Earlier studies have provided evidence of environmental toxins influencing sperm sex ratios (Kvist et al. 2014; Ullah et al. 2023; You et al. 2018), but the multigenerational implications of paternal exposure to these endocrine disruptors have not been investigated. Moreover, until now we did not understand the intergenerational nature of the sperm sex ratio under different social conditions. Uniquely, we have demonstrated that males have the potential to establish a legacy effect when it comes to influencing offspring sex; we found that fathers that experienced high‐male density conditions during sexual development led to their sons tending to produce greater proportions of daughter‐producing sperm. High‐male density may be a stressful condition that the fathers had to endure during development, and these marks may be imprinted in such a way that they are expressed in the subsequent generation. We acknowledge that the change we observed in sperm sex ratio between paternal treatments was, on average, small. However, we are excited about the prospect of more robust and extended discoveries with the use of different methodologies. Furthermore, we can only speculate on the underlying mechanism(s) that may lead to the intergenerational transmission of sperm sex ratio variation, and future investigation is required to confirm the potential mechanism that underlies our results. Producing daughters under high‐male density conditions is viewed as a parental strategy for reducing competition over resources (Shakes et al. 2011), but we do not yet understand how these inherited sperm sex ratios are reflected in different social landscapes experienced by the sons. In this sense, we do not yet have any clear information on the adaptive value of the intergenerational transmission of the sperm sex ratio. Our analyses, and in particular an effect size that may range from small to large, indicate that more rigorous testing is required to provide a more comprehensive understanding of the intergenerational nature of the sperm sex ratio. Our findings have opened up new and important pathways of discovery in the field of male‐driven sex allocation.

## Author Contributions

**Renée C. Firman:** conceptualization (lead), data curation (supporting), funding acquisition (equal), investigation (lead), methodology (lead), project administration (lead), resources (lead), supervision (lead), writing – original draft (lead), writing – review and editing (equal). **Francisco Garcia‐Gonzalez:** conceptualization (supporting), data curation (lead), formal analysis (lead), funding acquisition (equal), methodology (supporting), validation (lead), visualization (lead), writing – review and editing (equal).

## Conflicts of Interest

The authors declare no conflicts of interest.

## Supporting information


**Data S1:** ece372519‐sup‐0001‐DataS1.xlsx.

**Data S2:** ece372519‐sup‐0002‐DataS2.pdf.

**Figure S1:** Layout of the racks housing wild house mice that created the high‐male and high‐female environments. In the high‐male environment, ‘fathers’ (grey cells; numbers) were placed near sexually mature (blue cells; letters) and equivalently aged (blue cells; empty) nonfocal males. Twice a week, each ‘father’ was exposed to 15 g of soiled chaff from the 16 nonfocal sexually mature males (A–P). Once a fortnight each ‘father’ was released into a large, plastic opaque tub containing two of the sexually mature, nonfocal males (A–P). ‘Fathers’ were periodically exposed to soiled chaff from a sexually mature female to ensure normal reproductive development. In the high‐female environment, ‘fathers’ (grey cells; numbers) were placed near 16 sexually mature (pink cells; letters) and equivalently aged (pink cells; empty) females. Twice a week, each ‘father’ was exposed to 15 g of soiled chaff from the 16 sexually mature females (A–P). Once a fortnight each ‘father’ was released into a large, plastic opaque tub containing two of the sexually mature females (A–P). During the experiment, the ‘fathers’ were rotated across the different rack positions (within treatments).

## Data Availability

Data and underlying code are available as Supporting Information.
