# Supplementary material for: Does a Father's Social Environment Influence Their Sons' Sperm Sex Ratio? Potential for the Epigenetic Transmission of a Sex‐Allocating Mechanism
Source: Ecol Evol. 2025 Dec 26;15(12):e72519. doi: 10.1002/ece3.72519 (PMC12742443; doi:10.1002/ece3.72519)
Supplement: Supplementary file 3 — Figure S1: Layout of the racks housing wild house mice that created the high‐male and high‐female environments. In the high‐male environment, ‘fathers’ (grey cells; numbers) were placed near sexually mature (blue cells; letters) and equivalently aged (blue cells; empty) nonfocal males. Twice a week, each ‘father’ was exposed to 15 g of soiled chaff from the 16 nonfocal sexually mature males (A–P). Once a fortnight each ‘father’ was released into a large, plastic opaque tub containing two of the sexually mature, nonfocal males (A–P). ‘Fathers’ were periodically exposed to soiled chaff from a sexually mature female to ensure normal reproductive development. In the high‐female environment, ‘fathers’ (grey cells; numbers) were placed near 16 sexually mature (pink cells; letters) and equivalently aged (pink cells; empty) females. Twice a week, each ‘father’ was exposed to 15 g of soiled chaff from the 16 sexually mature females (A–P). Once a fortnight each ‘father’ was released into a large, plastic opaque tub containing two of the sexually mature females (A–P). During the experiment, the ‘fathers’ were rotated across the different rack positions (within treatments). [file ECE3-15-e72519-s003.docx]

**
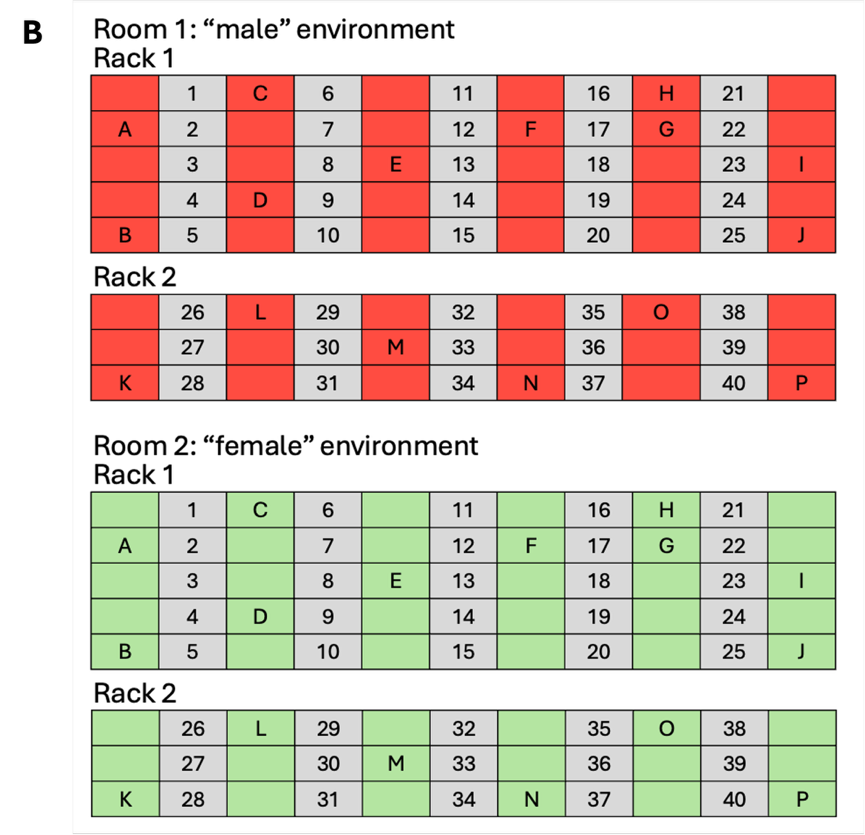
**

**FIGURE S1**⏐Layout of the racks housing wild house mice that created the high-male and high-female environments. In the high-male environment, “fathers” (grey cells; numbers) were placed near sexually mature (blue cells; letters) and equivalently aged (blue cells; empty) non-focal males. Twice a week, each “father” was exposed to 15 g of soiled chaff from the 16 non-focal sexually mature males (A – P). Once a fortnight each “father” was released into a large, plastic opaque tub containing two of the sexually mature, non-focal males (A – P). “Fathers” were periodically exposed to soiled chaff from a sexually mature female to ensure normal reproductive development. In the high-female environment, “fathers” (grey cells; numbers) were placed near 16 sexually mature (pink cells; letters) and equivalently aged (pink cells; empty) females. Twice a week, each “father” was exposed to 15 g of soiled chaff from the 16 sexually mature females (A – P). Once a fortnight each “father” was released into a large, plastic opaque tub containing two of the sexually mature females (A – P). During the experiment, the “fathers” were rotated across the different rack positions (within treatments).
